# Isotonic Glycerol and Sodium Hyaluronate Containing Artificial Tear Decreases Conjunctivochalasis after One and Three Months: A Self-Controlled, Unmasked Study

**DOI:** 10.1371/journal.pone.0132656

**Published:** 2015-07-14

**Authors:** Huba J. Kiss, János Németh

**Affiliations:** Semmelweis University, Department of Ophthalmology, Mária str. 39, H-1085, Budapest, Hungary; The Chinese University of Hong Kong, HONG KONG

## Abstract

Dry eye complaints are ranked as the most frequent symptoms of patients visiting ophthalmologists. Conjunctivochalasis is a common dry eye disorder, which can cause an unstable tear film and ocular discomfort. The severe conjunctivochalasis characterized by high LId-Parallel COnjunctival Folds (LIPCOF) degree usually requires surgical intervention, where a conservative therapy would be highly desirable. Here we examined the efficacy of a preservative-free, inorganic salt-free unit-dose artificial tear, called Conheal containing isotonic glycerol and 0.015% sodium hyaluronate in a prospective, unmasked, self-controlled study involving 20 patients. The regular use of the glycerol/hyaluronate artificial tear in three months caused a significant improvement in the recorded parameters. Conjunctivochalasis decreased from a mean LIPCOF degree of 2.9±0.4 on both eyes to 1.4±0.6 on the right (median decrease of -2 points, 95% CI from -2.0 to -1.0), and to 1.4±0.7 on the left eye (median decrease of -1 points, 95% CI from -2.0 to -1.0) (p<0.001 for both sides). The tear film breakup time (TFBUT) lengthened from 4.8±1.9 seconds on both eyes to 5.9±2.3 seconds (mean increase of 1.1 seconds, 95% CI from 0.2 to 2.0) and 5.7±1.8 seconds (mean increase of 0.9 seconds, 95% CI from 0.3 to 1.5) on the right and left eyes, respectively (p_right eyes_ = 0.020, p_left eyes_ = 0.004). The corneal lissamine staining (Oxford Scheme grade) was reduced from 1.3±0.6 on the right and 1.4±0.6 on the left eye significantly (p<0.001) to 0.3±0.4 and 0.2±0.4 on the right and the left eyes. The Ocular Surface Disease Index (OSDI) questionnaire score indicating the subjective complaints of the patients also decreased from a mean value of 36.2±25.3 to 15.6±16.7 (p<0.001). In this study, the artificial tear, Conheal decreased the grade of the conjunctivochalasis significantly after one month of regular use already, from the LIPCOF degree 3, considered as indication of conjunctival surgery, to a LIPCOF degree 2 or lower requiring a conservative therapy. Our results raise the possibility that vision-related quality of life can be significantly improved by conservative therapies even in severe conjunctivochalasis.

**Trial Registration:**

Controlled-Trials.com ISRCTN81112701 http://www.isrctn.com/ISRCTN81112701

## Introduction

Dry eye complaints occur in 5.5 to 33.7% of the population [1], and are ranked as the most frequent symptoms of patients visiting ophthalmologists [2–5]. Dry eye disease is caused by the reduced production and/or by the improper quality of the tear film. Evolution of dry eye disease may involve chronic inflammation of the ocular surface, and may lead to changes in the ocular surface. These changes may include injuries of the conjunctival and corneal epithelium, sagging of the conjunctiva, as well as the appearance of LId-Parallel COnjunctival Folds (LIPCOF). LIPCOF grading measures the number and severity of the lid-parallel conjunctival folds. LIPCOF grade 0 means the lack of the conjunctival folds, LIPCOF 1 signifies just one conjunctival fold, LIPCOF 2 stands for multiple conjunctival folds, not extending the tear meniscus, and LIPCOF 3 represents multiple conjunctival folds extending the tear meniscus. The LIPCOF degree correlates with the subjective dry eye symptoms [4, 6].

The severe conjunctivochalasis, characterized by the high LIPCOF degree, is also considered as a reason for the dry eye disease and not only as its consequence [7, 8]. The LIPCOF degrees show a strong correlation with both the subjective and objective complaints of the dry eye syndrome, as well as with the severity of the disease [9]. For all these reasons the assessment of LIPCOF degree changes in our study had by itself a great significance as a primary outcome measure of the study. However, tests studying the objective and subjective symptoms and functions of the ocular surface, should be evaluated together [10]. Therefore, in the course of our study, besides the changes in the LIPCOF degrees, we assessed the tear film breakup time (TFBUT) and the Oxford Scheme corneal staining [11] as well. The subjective complaints of our patients were recorded by the OSDI (Ocular Surface Disease Index), which is considered as the method easiest to follow [12]. These were the secondary outcome measures of our study.

In this examination we have assessed the effects of a unit-dose dose packaged, preservative-free, inorganic salt-free, isotonic glycerol- and 0.015% sodium hyaluronate-containing artificial tear, with special respect to its effect on conjunctivochalasis. Preliminary reports on the results of the one-month treatments of the current study was published in Hungarian language [13]. Our current results including both one and three-month treatments showed that conjunctivochalasis of LIPCOF degree 3, which is generally considered as indication for surgery [14], can be reduced by the help of a conservative therapy to a LIPCOF degree 2 or lower not requiring invasive therapy.

## Patients and Methods

### Patients

Twenty adult patients from the patients of the general outpatient unit of the Department of Ophthalmology of the Semmelweis University between 27^th^ August 2012 and 24^th^ July 2013 were enrolled into our prospective study approved by the Hungarian Scientific and Research-Ethics Committee (permission No. 21455-1/2011-EKU given on 7^th^ December 2011). The research followed the tenets of the Declaration of Helsinki. All participants gave their written informed consent to the examination. The trial was registered at the ISRCTN database (registration number: ISRCTN81112701) after the completion of the study, since trial database submission is not a compulsory in Hungary before starting such a single center study involving only a few patients. The authors confirm that all ongoing and related trials for this drug are registered. 16 female and 4 male patients participated in the study with a mean age of 64.0 ± 17.8 years (between 25 and 85 years of age). The complete date range of participant recruitment was between 27^th^ August 2012 and 24^th^ July 2013, and their follow-up studies were performed between 30^th^ August 2012 and 4^th^ November 2013. The number of patients was reduced from the originally planned 40 to 20, since power analysis of the first cohort of patients after a 1 month treatment showed a sufficient change of LIPCOF degree even after this short period. The examinations were finished at the Mária street section of the Department of Ophthalmology of the Semmelweis University, since the Tömő street section, mentioned as the study location in the study protocol, was moved to the Mária street section in the end of the study. The study was open, both patients and examiners knew the content of the eye drop.

Patient inclusion criteria were severe conjunctivochalasis (having LIPCOF degree 2 or higher) and lissamine green staining of minimum grade 1 or higher on the Oxford Scheme grade, indicating a more advanced dry eye disease. In the inclusion criteria we applied a more stringent criterion than the LIPCOF degree of 1 or higher in the study protocol, since we became interested, if the eye drops are also effective even in severe conjunctivochalasis conditions. In this severe condition the examination of personal satisfaction rate was omitted to reduce the examination time and patient stress and the examination of subjective symptoms was reduced to the self-completed OSDI questionnaire. Exclusion criteria included pregnancy or lactation, pterygium, prolonged treatment with eye drops with the exception of artificial tears, active allergic keratoconjunctivitis, current keratitis or conjunctivitis of infectious origin, surgery affecting the eye surface, as well as eye injuries occurred within 3 months before starting the treatment. There were no patients with tearing symptoms including punctal occlusion cases in this study. The patients have already used commercially available artificial tears regularly (17 out of the 20 for many months or years, 3 out of the 20 for a few weeks) before entering the study. Enrolled patients stopped the use of their earlier artificial tears 3 days before the first visit. On the first visit, inclusion criteria were re-checked, objective and subjective symptoms were recorded. No significant changes in either the objective or the subjective symptoms occurred during the 3 days wash-out period. A former related study [15] using hyaluronic acid containing artificial tear applied a 1-day wash-out period. This gives further support to our observation that 3 days are enough to overcome the potential delay effects of artificial tears.

### Description of treatments and examinations

Despite all of our dry eye patients have been continuously using artificial tears prior our study, they still had subjective symptoms, and their objective dry eye symptoms reached the advanced stage of LIPCOF 3. Since in spite of the use of the commercially available artificial tears each patient reached conjunctivochalasis LIPCOF grade 3 each patient served as his/her own control during the time of our study.

At the first visit the required amount of unit-doses of the preservative-free, inorganic salt-free artificial tear, Conheal (provided by Pannonpharma Ltd., Pécsvárad, Hungary), containing isotonic glycerol and 0.015% hyaluronic acid in purified water as described in reference [16] was given to our patients. Patients were instructed to apply these artificial tears on both eyes four times a day during the three months of the study. Due to the prior use of artificial tears and the detailed discussion of the study at the first visit, as well as the lack of serious adverse events of the eye drops used, patients' compliance was very high throughout the whole study as checked by discussions during the one-month and three-months-visits.

At the first visit, the best corrected visual acuity, the grade of conjunctivochalasis, the tear film breakup (TFBUT) time on both eyes was determined, and the extent of the epithelial damages of the cornea and conjunctiva with lissamine green staining. The subjective complaints of the patients, as well as the impact of the dry eye complaints on their everyday life were recorded by the help of the OSDI questionnaire [12]. Patients were asked to self-complete the OSDI questionnaire translated into Hungarian after receiving general instructions. Schirmer-tests and tear osmlolality measurements planned in the study protocol were not performed to reduce the invasiveness of the study and stress of patients having the advanced condition of severe conjunctivochalasis. After one and three months of regular use of the Conheal artificial tear, our patients were subjected to the same examinations.

The severity of the conjunctivochalasis was determined in terms of LIPCOF degrees according to the Höh method [17]. The TFBUT was measured by a standard method [18] using fluorescein. We opted for this standard method instead of the Tearscope examination planned in the original protocol, due to a sudden damage of our Tearscope apparatus at the start of the study causing inconsistency in its measurements, and since fluorescein staining of the eye was planned in the study protocol assessing epithelial damages besides lissamine green staining. The lissamine green staining was evaluated according to the Oxford Scheme grade [11].

To increase the validity of the measurements all measurements were performed by the same person during the whole study. The investigator was not aware of the stage of the patient, when performing the analysis. Measurements were supervised by an independent expert in a randomly selected 10% of the cases. Both the investigator and the independent expert had a Good Clinical Practice Certificate. LIPCOF degree measurements were performed on the same slit lamp having the same position and slit width in the whole study.

### Statistical evaluation

The results of the above tests recorded at the first visit were compared to the results of similar examinations after one and three month of treatment. Additionally, results after one month of treatment were compared to the results after three months of use of the artificial tears. For the comparison of ordinal data (LIPCOF degree, Oxford Scheme grade) and non-normally distributed data (OSDI) the non-parametric Wilcoxon Signed Rank Test was used, meanwhile the normally distributed data (TFBUT) were compared by the help of the parametric Paired T Test using the SPSS Statistics 22 software (IBM Corporation, Armonk, NY, USA). The statistical evaluation was refined compared to that planned in the study protocol including the evaluation of ordinal and non-normally distributed data. The results were expressed as mean ± standard deviation for each objective test and separately for the right and the left eyes. The OSDI test of course represents the personal satisfaction from the treatment of both eyes (Tables 1 and 2).

**Table 1:** Characteristic measures of the dry-eye syndrome after one month treatment with Conheal.

	Month 0*		Month 1*		Comparison Month 1 vs Month 0			
	mean±SD		mean±SD		Shift parameter** and 95% CI		P value***	
	right	left	right	left	right	left	right	left
LIPCOF degree	**2.9**±0.4	**2.9**±0.4	**1.8**±0.9	**1.6**±0.8	**-1.0** (-2.0 to -1.0)	**-1.0** (-2.0 to -1.0)	*<0*.*001*	*<0*.*001*
TFBUT value	**4.8**±1.9	**4.8**±1.9	**5.4**±1.6	**5.7**±2.1	**0.6** (0.2 to 1.0)	**0.9** (0.1 to 1.7)	*0*.*004*	*0*.*027*
Oxford grade	**1.3**±0.6	**1.4**±0.6	**0.6**±0.6	**0.6**±0.5	**-1.0** (-1.0 to 0.0)	**-1.0** (-1.0 to 0.0)	*<0*.*001*	*0*.*001*
OSDI Score	**36.2**±25.3		**22.6**±21.9		**-9.2** (-14.6 to -2.8)		*<0*.*001*	

*LIPCOF degrees, TFBUT values, Oxford Scheme grades and OSDI scores were measured, and statistical analysis was performed as described in Methods.

**This column contains the median difference and its nonparametric CI for LIPCOF, Oxford and OSDI (Corresponding to the Wilcoxon Signed Rank test), whereas it contains the mean difference and 95% CI based on the t-distribution for TFBUT.

***P-value of the Wilcoxon Signed Rank Test for LIPCOF, Oxford and OSDI, and P-value of the paired t-test for TFBUT (two-sided).

**Table 2:** Characteristic measures of the dry-eye syndrome after three months treatment with Conheal.

	Month 3*		Comparison Month 3 vs Month 0				Comparison Month 3 vs Month 1			
	Mean±SD		Shift parameter** and 95% CI		P value***		Shift parameter** and 95% CI		P value***	
	right	left	right	left	right	left	right	left	right	left
LIPCOF degree	**1.4**±0.6	**1.4**±0.7	**-2.0**(-2.0 to -1.0)	**-1.0**(-2.0 to -1.0)	*<0*.*001*	*<0*.*001*	**0.0**(-1.0 to 0.0)	**0.0**(0.0 to 0.0)	*0*.*035*	*0*.*312*
TFBUT value	**5.9**±2.3	**5.7**±1.8	**1.1**(0.2 to 2.0)	**0.9**(0.3 to 1.5)	*0*.*020*	*0*.*004*	**0.5**(-0.3 to 1.3)	**-0.0**(-0.7 to 0.6)	*0*.*191*	*0*.*953*
Oxford grade	**0.3**±0.4	**0.2**±0.4	**-1.0**(-1.0 to -1.0)	**-1.0**(-1.0 to -1.0)	*<0*.*001*	*<0*.*001*	**0.0**(-1.0 to 0.0)	**0.0**(-1.0 to 0.0)	*0*.*016*	*0*.*039*
OSDI Score	**15.6**±16.7		**-13.1**(-25.0 to -8.3)		*<0*.*001*		**-2.4**(-5.7 to 0.0)		*0*.*012*	

*LIPCOF degrees, TFBUT values, Oxford Scheme grades and OSDI scores were measured, and statistical analysis was performed as described in Methods. For Month 0 and Month 1 values, see Table 1.

**This column contains the median difference and its nonparametric CI for LIPCOF, Oxford and OSDI (Corresponding to the Wilcoxon Signed Rank test), whereas it contains the mean difference and 95% CI based on the t-distribution for TFBUT

***P-value of the Wilcoxon Signed Rank Test for LIPCOF, Oxford and OSDI, and P-value of the paired t-test for TFBUT (two-sided). Gray boxes represent the non-significant changes.

To study the influence of sample size reduction from 40 to 20 subjects in the study, a post hoc power analysis was performed for the primary efficacy outcome measure, mean grade of conjunctivochalasis.

For a conservative power estimation the highest observed within-group standard deviation of 0.9 (Tables 1 and 2) was supposed, and a low correlation value of 0.2 was assumed for correlated measurements performed on the same subject at month 0 and month 3. A mean decrease from baseline of 1.0 LIPCOF degree was assumed to be clinically relevant. Power estimation was performed for a paired t-test at a two-sided significance level of 5%, and corrected for the asymptotic relative efficiency of 3/π because during the statistical evaluation the Wilcoxon Signed Rank Test was applied instead. Power calculations were performed in SAS v9.4.

For the assumptions described above the power to show a clinically relevant mean decrease of 1 degree in the primary outcome measure was 95.1%. Since this was an exploratory study no adjustment for multiplicity was made, although the primary outcome measure was tested both at the right and the left eye.

## Results

**Fig 1:**
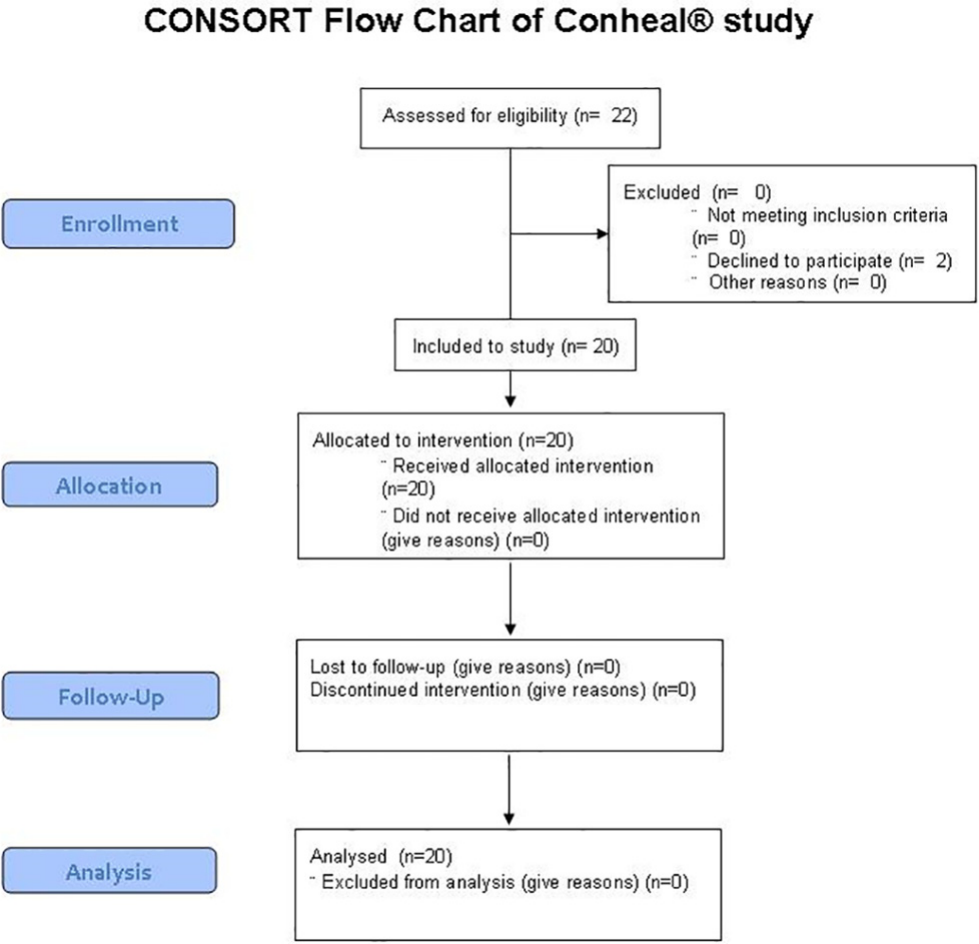
CONSORT flowchart of the study. The figure shows the CONSORT flowchart of the study. The TREND checklist (S1 TREND Checklist) and the protocol of the study (S1 Protocol) can be found as supporting information files.

**Fig 2:**
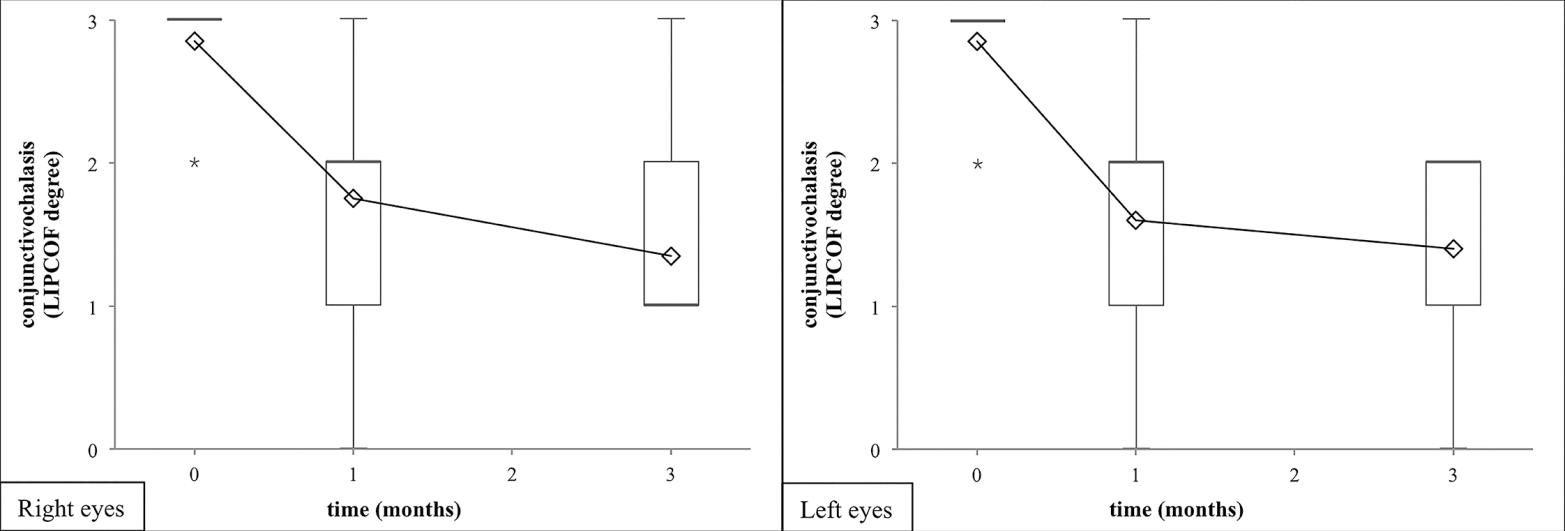
Degree of the conjunctivochalasis in terms of LIPCOF degrees after 1 and 3 months of artificial tear treatment. Artificial tear treatment and measurement of LIPCOF degree on 20 patients were performed as described in Methods. The artificial tear investigated caused a significant decrease of LIPCOF degree on both eyes after one month of use that advanced further on the right eye (filled diamonds, solid line) significantly, and showed the same tendency on the left eye (open rectangles, dashed line) after three months of use. Means and their standard errors of the LIPCOF degree are shown. Statistical evaluation was performed using the Wilcoxon Signed Rank Test. One and three asterisks note p<0.05 and p<0.001, respectively.

Fig 1 shows the CONSORT flow chart of the study. The TREND checklist (S1 TREND Checklist) and the protocol of the study (S1 Protocol) can be found as supporting information files. Study details are given in the Methods section. After both one and three months of properly scheduled treatments with Conheal, regularly instilled four times a day, both the subjective and objective symptoms of our patients improved. The numerical results of the examinations performed at the first visit (starting-visit), after one month (one-month-visit) and three months of use (three-month-visit) of the artificial tears are summarized in Tables 1 and 2. The primary outcome measure of our study, the mean grade of the conjunctivochalasis was reduced on both eyes significantly at the one-month-visit (Fig 2, Table 1), and was decreased further at the three-month-visit (Fig 2, Table 2). After three months conjunctivochalasis decreased from a mean LIPCOF degree of 2.9±0.4 on both eyes to 1.4±0.6 on the right (median decrease of -2 points, 95% CI from -2.0 to -1.0), and to 1.4±0.7 on the left eye (median decrease of -1 points, 95% CI from -2.0 to -1.0) (p<0.001 for both sides).

**Fig 3:**
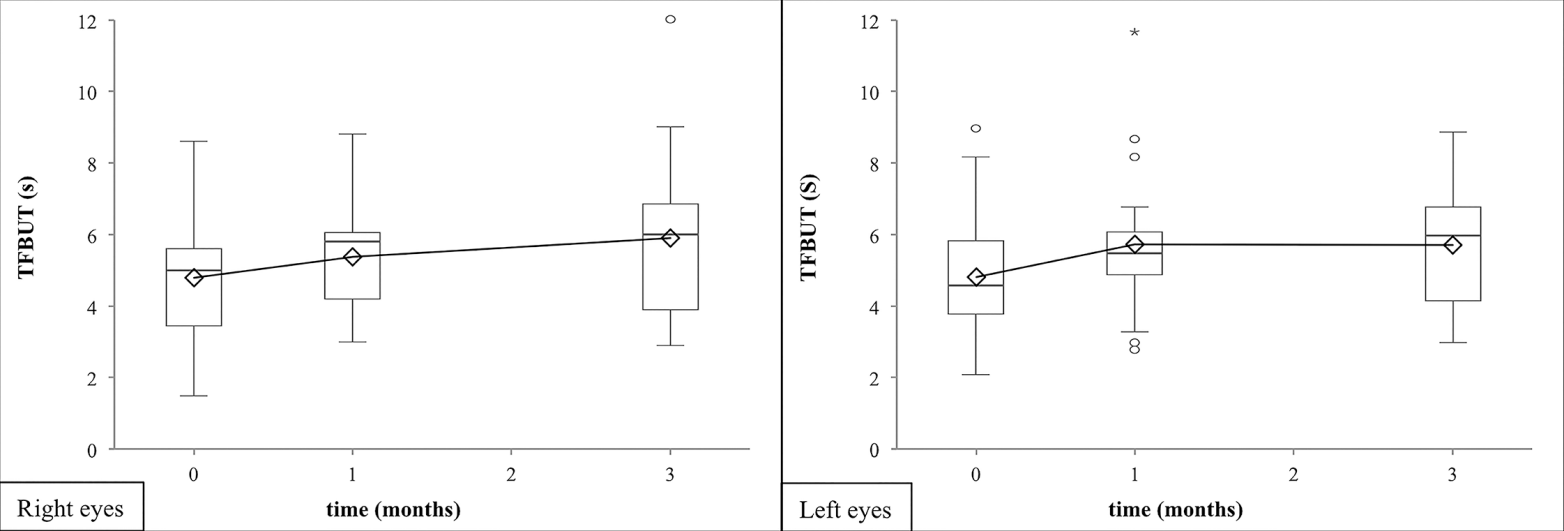
Tear-film breakup time after 1 and 3 months of artificial tear treatment. Artificial tear treatment and measurement of TFBUT on 20 patients were performed as described in Methods. Tear-film breakup time (TFBUT) showed a significant elongation after one month of regular use of the artificial tears, the effect did not grow further with the longer treatment both on right and left eyes (shown by filled diamonds/solid line, or open rectangles/dashed line, respectively). Means and their standard deviations of the TFBUT value are shown. Statistical evaluation was performed using the Paired T Test. One and two asterisks note p<0.05 or p<0.01, respectively.

**Fig 4:**
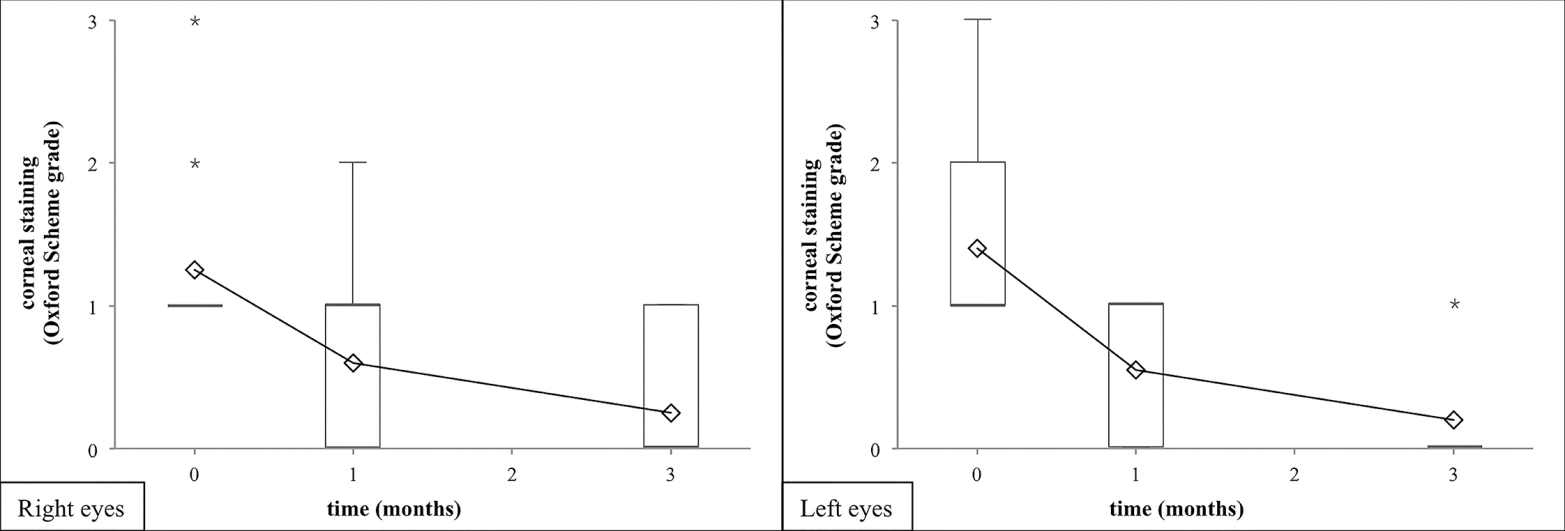
Lissamine staining after 1 and 3 months of artificial tear treatment. Artificial tear treatment and measurement of lissamine staining (in terms of the Oxford Scheme grade) on 20 patients were performed as described in Methods. Lissamine staining significantly decreased on both eyes (right eyes: filled diamonds/solid line, left eyes: open rectangles/dashed line) after one and three months of treatment with the artificial tear product. Means and their standard errors of the Oxford Scheme grade are shown. Statistical evaluation was performed using the Wilcoxon Signed Rank Test. One, two and three asterisks note p<0.05, p<0.01 and p<0.001, respectively.

The other two recorded objective secondary outcome measures of the dry eye disease also improved during our study. The tear film breakup time (TFBUT) lengthened till the one-months-visit (right eye median increase of 0.6 sec, 95% CI from 0.2 to 1.0 sec; left eye median increase of 0.9 sec, 95% CI from 0.1 to 1.7 sec), but no significant increase was found after. (Fig 3, Tables 1 and 2). By the application of lissamine green staining the mean Oxford Scheme grade staining decreased significantly during the entire period of the examination (right eye median decrease of -1.0 grade, 95% CI from -1.0 to -1.0 grade; left eye median decrease of -1.0 grade, 95% CI from -1.0 to -1.0 grade after three months; Fig 4, Tables 1 and 2). There were no significant differences between the results of the objective tests on the right and the left eyes (p>0.050).

**Fig 5:**
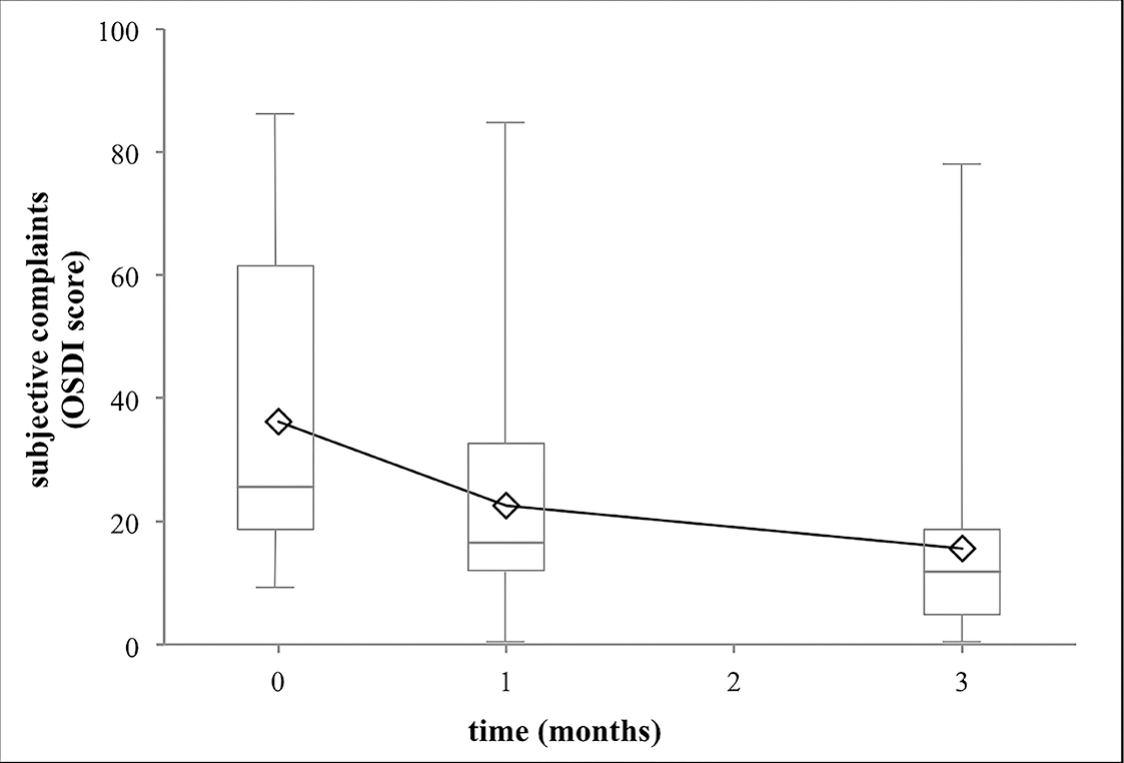
Impact of 1 and 3 months of artificial tear treatment on the subjective complaints of patients. Artificial tear treatment and measurement of OSDI score on 20 patients were performed as described in Methods. The OSDI scores showed a significant decrease at the end of the first month, which decreased further significantly at the end of the third month. Means and their standard errors of the OSDI score are shown. Statistical evaluation was performed using the Wilcoxon Signed Rank Test. One and three asterisks note p<0.05 and p<0.001, respectively.

The subjective complaints of the patients measured with the Ocular Surface Disease Index (OSDI) questionnaire as the third secondary outcome measure of the study showed a significant improvement during three months (median decrease of -13.1 scores, 95% CI from -25.0 to -8.3 scores; Fig 5, Tables 1 and 2). The high standard deviation was caused by the broad spectrum of the OSDI data, since the subjective complaints showed a high individual scatter. In all of our patients a decrease in the OSDI values could be observed at the end of the three months examination, the range of the extent of the decrease was from 2.1 to 61.7 OSDI scores.

We have also evaluated the changes of LIPCOF degrees and OSDI scores in separate groups of patients having different initial LIPCOF degrees (14 patients having an initial LIPCOF degree 3 on both eyes, 6 patients having an initial LIPCOF degree 3 on one and LIPCOF degree 2 on the other eye. No patients had LIPCOF degree 2 or lower on both eyes).

Only one (2.94%) out of the 34 eyes that had LIPCOF degree 3 conjunctivochalasis initially had an unchanged LIPCOF degree 3 during the study. LIPCOF degree decreased from the initial degree 3 to degree 2 or degree 1 in 44.1% or 47.1% of the eyes, respectively. LIPCOF degree 0 was reached in two eyes (5.9%). The conjunctivochalasis decreased to degree 1 in 100% of the 6 eyes having LIPCOF degree 2 conjunctivochalasis at the beginning of the study.

The OSDI score decreased in both patient groups having a bilateral LIPCOF degree 3 or a LIPCOF degree 2 and 3 in different eyes initially. The average OSDI score of the group having bilateral LIPCOF degree 3 conjunctivochalasis decreased from the initial value of 29.6 to 20.5 at the end of the first month and to 15.9 at the end of the three months. The OSDI score was surprisingly higher at the beginning of the study in the group having unilateral LIPCOF degree 3, and LIPCOF degree 2 on the other eye, but the OSDI score decreased dramatically from the initial value of 51.5 to 27.5 at the end of the first month and to 14.8 at the end of the three months. At the end of the three months trial no significant difference was found between the results of the two groups (p = 0.304).

During the study period two patients complained about greasy sensation on the eyelids, but they did not stop the treatment. No other adverse reactions were reported.

## Discussion

In the course of our study the preservative-free, inorganic salt-free, isotonic glycerol and 0.015% sodium hyaluronate containing Conheal artificial tears administered four times a day resulted in a significant favorable change on the ocular surface after one month of regular use already that progressed during the three months of the examination. The treatment of the patients, who used other commercially available artificial tears earlier, resulted in improved objective symptoms and increased patients’ subjective satisfaction.

From our results, the effect of the artificial tears on the degree of conjunctivochalasis, which was the main purpose of the study and is the main point of our paper, is a novel finding in the literature. Decrease of LIPCOF degree has been reported earlier in the evaporative dry eye disease after the treatment with liposome eye spray [19–22]. However, this study is the first demonstrating the decrease of conjunctivochalasis of patients suffering from keratoconjunctivitis sicca using eye drops not containing lipids. This result is important, since it demonstrates the existence of a conservative therapy of severe conjunctivochalasis leading to the reduction of the LIPCOF degree. Using the artificial tears applied in this study, the LIPCOF degree 3, considered as indication for surgery [14], became controllable, and resulted in less corneal damage, hence less complaints.

Our results are in agreement with the results of an earlier clinical study performed with an artificial tear preparation of identical composition. In that article they also showed, that the improving of the rose bengal staining lead to improved personal satisfaction. However, the earlier report did not measured the extent of conjunctivochalasis [16], which is then major novelty of the current paper. In agreement with earlier studies carried out with sodium hyaluronate containing products, the tear film breakup times were lengthened [23], and the epithelial damages were resolved due to the reduction of chronic harms of the corneal epithelium [24]. In agreement with our results, the prolongation of the tear film breakup time was recorded with glycerol containing artificial tears earlier [25]. The subjective complaints of the patients measured with the OSDI questionnaire [12], ensuring a good follow-up, have decreased in our study, which is in agreement with the general correlation between OSDI data and the grade of conjunctivochalasis described by Németh et al. [9].

It is known from the literature that in conjunctivochalasis of severe grade showing high lissamine green staining, the human leukocyte antigen-DR (HLA-DR) level is elevated [26]. Thus it is possible that Conheal caused a decrease in the severity of the conjunctivochalasis through lowering the HLA-DR level, since in keratinocytes glycerol decreases the toll-like receptor 2 (TLR2) and TLR3 activation caused upregulation of the expression of HLA-DR [27]. It is important to mention that other authors found that sodium hyaluronate containing eye drops were able to increase the TFBUT [23, 28] and induced a decrease in corneal staining [29, 30]. The eye drops used in our study contained a combination of glycerol and sodium hyaluronate, and caused significant improvement in both the objective and subjective symptoms of the patients. In light of the results of previous studies [31, 32] and in light that in conjunctivochalasis the HLA-DR is increased [26], it is likely that during our study the HLA-DR levels decreased without the use of a corticosteroid [31] or other anti-inflammatory agent [33]. A tear proteomics study of conjunctivochalasis patients highlighted apoptosis- and inflammation-related proteins, as well as an increased level of tear defensin associated with conjunctivochalasis [34]. Further studies are needed to ascertain, whether these changes were induced as a consequence of the severe condition, and/or were also contributing to the etiology of conjunctivochalasis.

Hyaluronic acid retains water on the ocular surface, and improves lubrication [35]. These effects (together with the similar effects of glycerol) may have contributed to the overall efficiency of the eye drops used besides potential specific mechanisms, such as attenuation of the upregulation of HLA-DR, as well as apoptosis- and inflammation-related proteins.

**Fig 6:**
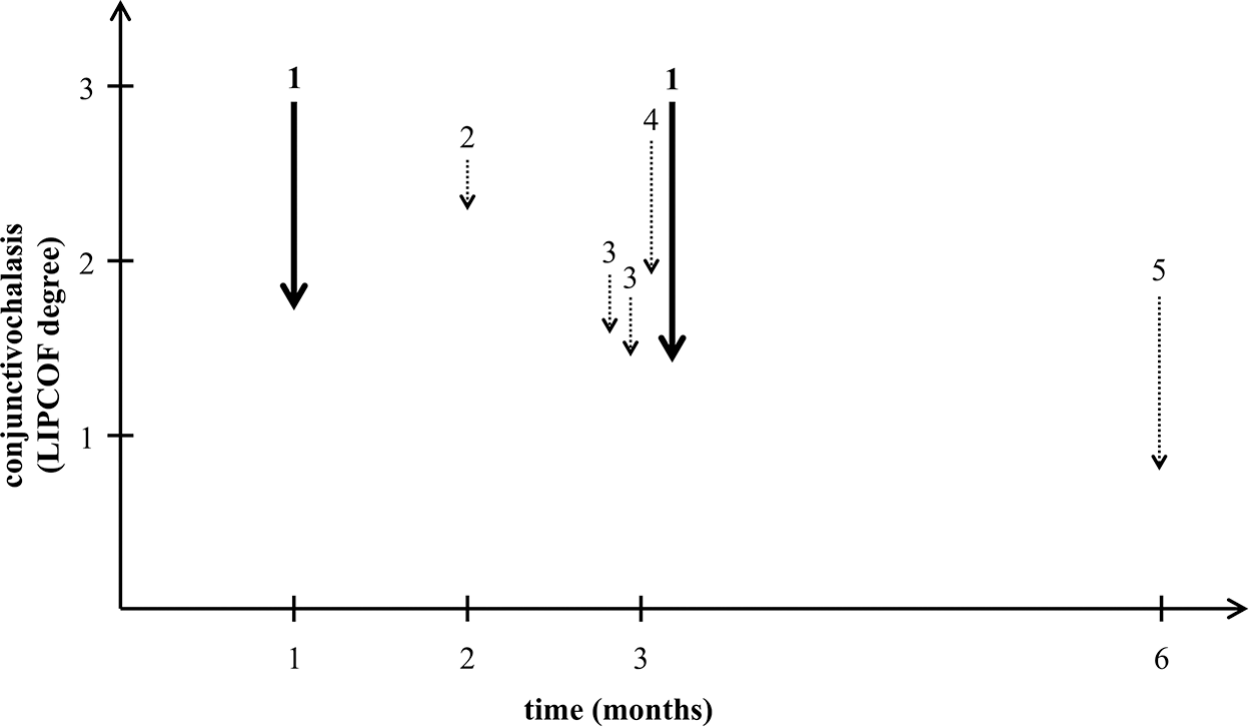
Comparative analysis of the decrease of conjunctivochalasis in various studies. The figure shows the decrease of the degree of conjunctivochalasis compared to that measure at the start of the treatment in previous studies using a liposome eye spray (dotted arrows) compared to our results using an isotonic glycerol and 0.015% hyaluronate-containing artificial tear (solid lines). Numbers above the arrows represent the different studies on a time scale of their duration in months. 1: current results, 2: Höh et al. [21], 3: group 1 and 2 of Dausch et al. [20], 4: Khaireddin et al. [22], 5: Lee et al. [23].

The separate use of both glycerol and sodium hyaluronate in the artificial tear preparations is known for a long time, and was shown to be safe [29, 35, 36]. The effect of glycerol [37] and hyaluronic acid [23, 28] treatments alone induced the prolongation of the non-invasive tear film breakup time (NIBUT), and the healing of epithelium injuries developed as a consequence of the dry eye disease [24]. A decrease of the LIPCOF degree has been demonstrated on evaporative dry eyes in several studies using a liposome eye spray [19–22]. In those studies the dry eye patients were diagnosed primarily by the tear-film instability, not the conjunctivochalasis. However, the decrease of the LIPCOF degree observed in those studies was less than observed in our study using a lipid-free artificial tear containing isotonic glycerol and 0.015% hyaluronic acid (Fig 6). The liposome eye spray was efficient in the increasing of the TFBUT and decreasing the lid-margin inflammation [19–22].

The administration of preservative-free preparations is generally more beneficial than the use of the products containing even recently applied preservatives [38]. The use of unit-dose packaged artificial tears is both simple and safe, since they do not become infected within 24 hours after opening [39]. However, the infection-free period has to be determined for every unit-dose preparation individually, as it was done for Conheal resulting in 12 hours of infection-free period.

The primary treatment of the dry eye syndrome is the chronic administration of artificial tears. However in the case of severe conjunctivochalasis (LIPCOF degree 3), an invasive therapy may also be necessary [14]. A spectrum of invasive therapies, like classical surgeries against conjunctivochalasis and other invasive methods, such as the treatment of the conjunctival folds with argon-laser [40] or with heat cauterization [41], are well known.

There is little data on population survey of conjunctivochalasis. A study in Shanghai [42] claims that over 60 years 4% of the people had “very severe” (grade 3) conjunctivochalasis (at least 0.25% needing urgent surgery) and 16% had “severe” (grade 2) conjunctivochalasis, therefore grade 2 and grade 3 conjunctivochalasis occurred in about 20% of the population over the age of 60. In the EU about 20% of the people are over the age of 65 [43]. From these data it might be assumed that in the total EU population 0.8% has grade 3 conjunctivochalasis (approximately 0.03% needing urgent surgery) and 3.2% have grade 2 conjunctivochalasis: in total 4%.

Since dry eye complaints occur in 5.5 to 33.7% of the population [1] (having an average prevalence of 10.3% weighted to the number of patients participated in the study) we might assume that the incidence of the conjunctivochalasis-caused dry eye syndrome in the population is about one-third of all dry eye cases.

All of our patients used various artificial tears regularly, which did not alleviate their symptoms and in spite of their regular use the patients’ LIPCOF degree raised to a mean of 2.9±0.4. The lack of the satisfactory effect of the previous therapies on the objective and subjective symptoms of dry eye disease made our examinations a self-controlled study. The power analysis showed that the study was adequately powered (power>95%) even after reducing the sample size from 40 to 20 patients.

In conclusion, the artificial tear, Conheal, decreased the grade of the conjunctivochalasis significantly after one month of regular use already, from the LIPCOF degree 3, considered as indication of conjunctival surgery, to a LIPCOF degree 2 or lower requiring a conservative therapy. Our results raise the possibility that vision-related quality of life can be significantly improved by conservative therapies even in severe conjunctivochalasis.

## Supporting Information

S1 TREND Checklist TREND checklist. TREND Statement Checklist for the trial.
(PDF)

S1 Protocol Study protocol. Study protocol for the examination of the efficiency of Conheal sodium-hyaluronate containing eye drops in conjunctival and corneal epithelial injuries as approved by the Hungarian Scientific and Research-Ethics Committee (permission No. 21455-1/2011-EKU).
(PDF)
