# Supplementary material for: Isotonic Glycerol and Sodium Hyaluronate Containing Artificial Tear Decreases Conjunctivochalasis after One and Three Months: A Self-Controlled, Unmasked Study
Source: PLoS One. 2015 Jul 14;10(7):e0132656. doi: 10.1371/journal.pone.0132656 (PMC4501551; doi:10.1371/journal.pone.0132656)
Supplement: S1 Protocol — Study protocol for the examination of the efficiency of Conheal sodium-hyaluronate containing eye drops in conjunctival and corneal epithelial injuries as approved by the Hungarian Scientific and Research-Ethics Committee (permission No. 21455-1/2011-EKU). (PDF) [file pone.0132656.s002.pdf]

English translation of the study protocol for the examination of the efficiency of ConHeal® sodium-hyaluronate containing eye drops in conjunctival and corneal epithelial injuries

Approved by the Hungarian Scientific and Research-Ethics Committee
(permission No. 21455-1/2011-EKU).

The experimental preparation:

0.15 mg/ml sodium-hyaluronate
glycerol, Carbomer 981

Package
As sterile eye drops prepared by Pannonpharma Co.

Inclusion of patients:

a) approximately 40 persons
b) recruited from the patients of the general outpatient unit of the Department of Ophthalmology of the Semmelweis University

Inclusion criteria:

a) volunteer above 18 years of age
b) having a LIPCOF degree of 1 or higher
c) having an epithelial injury of I. or higher in the Oxford scale measured by lissamine green staining
d) having a 3rd or worse personal satisfaction rate

Exclusion criteria:

a) pregnancy, lactation
b) pterygium
c) prolonged treatment with eye drops

d) active allergic keratoconjunctivitis

e) current keratitis or conjunctivitis of infectious origin

f) surgery affecting the eye surface, as well as eye injuries occurred within 3 months before starting the treatment.

Study protocol:

Inclusion (0. visit):

On the first examination of the patients believed to be includable to the study the eye status-related comfort level of the patient is examined by a Personal Satisfaction test having 1 to 5 grades and using schematic human faces showing different levels of satisfaction. We also determine the level of the eye surface epithelial injury using lissamine green staining and the Oxford Scheme grade (Bron AJ, 2003). We will include to the study patients having degree 3 or worse personal satisfaction and having a lissamine green staining of grade I. or higher.

Examination I.:

We interview the patient on the eye on the nature of her/his eye-related complaints (eye dryness, burning, watering, redness, fatigue and extraneous body feeling), as well as the degree and prevalence of the complaints (OSDI questionnaire and Personal Satisfaction test). Patients potentially enrolled to the study undergo a general ophthalmological examination including uncorrected and best corrected long-distance visual acuity, the potentially required eye-glasses correction, as well as the slit lamp examination of the anterior segment of the eye. We also perform the examination of the LId-Parallel COnjunctival Folds (LIPCOF), and we assess their severity on a 4-degree scale. We also assess the tear film breakup time (TFBUT). For these measurements we will use Tearscope Plus® tear film examination device, where we examine the reflection of the grid of the device on the cornea and we measure the tear film breakup time as the time passed between twinkling and the distortion of the reflected pattern. We determine the epithelial damages of the cornea and conjunctiva using fluoresceine and/or lissamine green staining on the Oxford Scale. We measure the osmolality of the tear using a TearLab® osmometer (by immersion of the sterile, single-use examination headpiece to the tear meniscus at the side of the eyelid). The extent of tear production will be measured using the Schirmer-test, where we measure the amount of tear produced in 5 minutes by placing a sterile filter paper to the bottom conjunctival sac. Using the results of these examination we determine the level of tear film dysfunction on the „Dry Eye Severity” scale.

Enrolled patients will receive the ConHeal eye drops. The study is open, both patients

and examiners will know the content of the eye drop. The periodicity of, as well as the proper way of the eye drop application will be discussed with the enrolled patients in detail.

Examinations II / III:

We repeat the examinations of Examination I. and we explore all comments in connection with the application of the eye drop, specifically asking about subjective changes and potential side effects.

These examinations will be performed on the 28th to 30th, as well as 80th to 90th days of the treatment, respectively.

The planned examination methods are widely used and accepted in the international and Hungarian ophthalmological practice. The examinations and sample collections of the study will be performed at the Tömő street section of the Department of Ophthalmology of the Semmelweis University.

Information of patients and written informed consent

The patient will be informed on the aim of the study, on the circumstances and protocol of measurements. Data of examinations will be recorded on a separate page where the name of the patient is not included only a randomly generated code/number.

Assessment of statistical analysis:

At the end of the study we will examine the data using non-parametric t-probes and correlation tests.
